# Diffusion-Driven Looping Provides a Consistent Framework for Chromatin Organization

**DOI:** 10.1371/journal.pone.0012218

**Published:** 2010-08-25

**Authors:** Manfred Bohn, Dieter W. Heermann

**Affiliations:** 1 Institute for Theoretical Physics, Heidelberg University, Heidelberg, Germany; 2 The Jackson Laboratory, Institute for Molecular Biophysics, Bar Harbor, Maine, United States of America; King Abdullah University of Science and Technology, Saudi Arabia

## Abstract

Chromatin folding inside the interphase nucleus of eukaryotic cells is done on multiple scales of length and time. Despite recent progress in understanding the folding motifs of chromatin, the higher-order structure still remains elusive. Various experimental studies reveal a tight connection between genome folding and function. Chromosomes fold into a confined subspace of the nucleus and form distinct territories. Chromatin looping seems to play a dominant role both in transcriptional regulation as well as in chromatin organization and has been assumed to be mediated by long-range interactions in many polymer models. However, it remains a crucial question which mechanisms are necessary to make two chromatin regions become co-located, i.e. have them in spatial proximity. We demonstrate that the formation of loops can be accomplished solely on the basis of diffusional motion. The probabilistic nature of temporary contacts mimics the effects of proteins, e.g. transcription factors, in the solvent. We establish testable quantitative predictions by deriving scale-independent measures for comparison to experimental data. In this Dynamic Loop (DL) model, the co-localization probability of distant elements is strongly increased compared to linear non-looping chains. The model correctly describes folding into a confined space as well as the experimentally observed cell-to-cell variation. Most importantly, at biological densities, model chromosomes occupy distinct territories showing less inter-chromosomal contacts than linear chains. Thus, dynamic diffusion-based looping, i.e. gene co-localization, provides a consistent framework for chromatin organization in eukaryotic interphase nuclei.

## Introduction

The cell nucleus is a main constituent of eukaryotic organisms and yet its complexity prevents detailed knowledge of its function. The genome content is carried by the chromosomes: compactly folded polymers consisting of DNA and histone proteins. While during mitosis chromosomes are found in an extremely condensed state, the chromatin fiber inside the interphase nucleus has a much more decondensed organization. However, at this stage of the cell cycle, highly coordinated processes such as transcription, replication and DNA repair take place, making a random folding of the chromatin fiber very unlikely. A pivotal question is the connection between genome organization and function, which could not be answered in a satisfying way up to now. The organization of the genome in the interphase nucleus of eukaryotic cells is done on multiple scales of length and degrees of compaction. The basic filament is the DNA double helix which is wrapped around histone cores forming the nucleosome. The chromatin fiber is a complex of nucleosomes and linker DNA forming a beads-on-a-string type of filament with a diameter of about 11 nm [1]. In-vitro experiments provide evidence that this structure in turn condenses under certain salt conditions to an even more compact structure of 30 nm, but both its regularity and its existence in living cell nuclei are still under debate [2]–[5]. Stunningly, even less is known about the structural organization on a scale above 30 nm. Up to now, experimental techniques are limited by the resolution of conventional light microscopes of about 200 nm, requiring indirect assays for investigating chromatin folding. Several experimental techniques have been applied: Fluorescent labeling of large parts of a chromosome yields results on structure, shape and position of chromosomal regions [6] or even of entire individual chromosomes [7]. Labeling two loci of a chromosome with a fluorescent marker was successfully used to establish a relationship between genomic distance 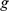 between these markers and its mean square physical distance in yeast [8], drosophila [9], [10] and human cells [11], [12].

There is now abundant evidence that genome function is tightly related to chromatin folding on several length scales. The one-dimensional distribution of genes along the chromosome is far from being random: the Human Transcriptome Map [13] reveals a clustering of active genes as well as inactive genes into certain domains, which have been named ridges and anti-ridges [6]. Various experiments have shown that the 3D organization of chromatin depends on transcriptional activity: Active genes tend to be located in the nuclear interior while inactive genes are found more often at the nuclear periphery [6], [14], [15], the converse behavior is observed in some experiments [16]. Moreover, a change in the transcriptional state of a gene can have direct influence on its positioning inside the nucleus [17], [18]. Transcriptional active regions (ridges) were observed to have a more open structure than inactive regions (anti-ridges) [6]. Also, the relationship between mean square distance (MSD) of two fluorescent markers and their genomic separation has revealed significant differences in compaction between ridges and anti-ridges [12]. Further, these fluorescence in situ hybridization (FISH) experiments displayed a leveling-off in the MSD for genomic separations above 10 Mb (mega basepairs), the plateau level being in the size range of 2 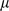
m. On the scale of the nucleus, chromosomes are separated into distinct chromosome territories [7], whose relative positions and ellipsoidal shape varies from cell to cell [19].

Intra-chromosomal as well as inter-chromosomal contacts or loops have been intensively analyzed in the past few years both experimentally and theoretically as a possible mechanism for transcriptional regulation and genome folding. Yet, chromatin loops seem to be an ubiquitous feature of genome organization and genome function. Transcriptional regulation is often controlled by regulatory motifs such as enhancers and silencers. These can be located tens of kb apart from the target gene which they regulate [20], [21]. One possible interaction mechanism is spatial proximity of regulatory element and target gene which requires the looping out of intervening DNA. 3C experiments have demonstrated that this is indeed the case in the 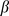-globin locus [22]. One idea put forward to explain chromatin loops is the existence of transcription factories or active chromatin hubs, where active polymerases cluster and thereby co-locate genes and regulatory elements [23], [24]. 3C and 4C techniques have since then provided evidence that indeed loops up to several Mb exist in interphase cells [20], [25]. However, the detailed mechanisms and driving forces of looping are still under debate.

We present a polymer model, the Dynamic Loop model, where functional loops are formed solely on the basis of diffusional motion. Importantly, loops are assumed to be dynamic and the sets of loop attachment points change during time. Thus, our loop model is minimal meaning that we do not assume a priori long-range forces and active transport mechanisms. Besides the new motif of dynamic loop formation, this is a major advance with respect to other chromatin models with loops [11], [12], [26]–[29]. Various other polymer models have been proposed [30], [31] which do not take into account chromatin looping. The assumptions of our model arise from biological evidence: 4C experiments clearly show that loops exist on length scales from several thousand basepairs to tens of Mb [25]. Surely, if looping is related to functional processes like transcriptional regulation and the formation of transcription factories, the cell must be able to control this looping dynamically. Large cell-to-cell variations in FISH distance measurements [12], [32] render such a dynamics a necessary feature of any polymer model.

Our model makes testable predictions on a variety of observable quantities. We predict that chromosomes fold into a confined space and display a different fluctuation regime than non-dynamic looping polymers or linear chains. Importantly, the formation of large loops can be accomplished hierarchically mediated by many loops on the short scale without the assumption of long-range interactions. We demonstrate that the beads of the polymer display sub-diffusive behavior in agreement with experimental data [17] and that chromosome territories are constituted driven by looping; the overlap between different chromosome territories (CTs) depends on the local looping probabilities.

### The chromatin model

Our model starts by initially assuming chromatin to consist of a coarse-grained linear polymer chain. Loop formation is achieved on the basis of diffusional motion of the monomers in the following way: Whenever two segments co-localize by diffusional motion, a chromatin loop is formed with a certain probability 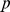 between these two sites. A certain lifetime is assigned to each loop, thus loop attachment points dissolve again during the course of time. Lacking experimental knowledge on the time scales over which chromatin segments remain co-localized, e.g. in transcription factories, different looping lifetimes are considered. Details are described in Materials & Methods.

The stochastic nature of loop formation provides method to effectively incorporate protein-chromatin and chromatin-chromatin interactions. Looping is often thought to be mediated by DNA-binding factors such as CTCF [33], Sat1B [34] or PcG [10] or by regions of increased polymerase concentration, i.e. transcription factories [24]. The probabilistic creation of functional chromatin contacts mimics the effect of protein concentration (there being either proteins binding DNA sites or not) and binding affinity. The detailed nature of the binding affinity thus does not need to be considered explicitely in our model. In the following we denote by “loop” a functional interaction between two parts of a chromatin fiber existing for a certain time as created by the algorithm. In contrast, a “contact” denotes two parts of the chromatin fiber close together by thermal fluctuations without necessarily being an interaction.

A typical human chromosome has a length of about 100 mega basepairs (Mb), rendering a detailed description on the molecular level computationally impossible. Typically, coarse-grained approaches are used, where a long stretch of chromatin is modeled as an effective monomer. Polymer scaling theory [35] tells us that for linear polymers such an approach is well justified above the scale where bending rigidity plays a role. This length scale is established by the persistence length 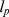, defining the transition from a rod-like to a flexbile polymer. Estimates for the persistence length range from 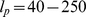 nm [36] but are often based on crude approximations by fitting data to linear chain models [8], [30]. Thus, it is reasonable to conduct computer simulations on a coarse-grained scale where it can be securely assumed that the fiber is flexible.

To study the impact of diffusion-based loop formation on the conformational properties isolated from effects of the presence of other chromosomes, we simulate single chromosomes in a dilute solution. In fact, it has been argued that the disentanglement time for the transition from interphase to metaphase chromosomes of size 100 Mb is in the order of 500 years [37], [38], thus requiring the activity of DNA topoisomerase II. Rosa *et al.* reversed the argument proposing that interphase chromosomes never equilibrate [38]. We ask whether the observed confined folding already arises from the experimentally confirmed loop formation without invoking rather unprecise knowledge of time and length scales. If loop formation turns out to cause confined folding, then the presence of other chromosomes should not alter the conformational properties drastically. That is why we focus first on isolated chains. In a coarse-grained approach we study chain lengths of size 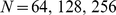 and 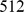. We use Monte-Carlo simulations on a cubic lattice employing the well-established bond fluctuation algorithm [39]. The lattice size is chosen to be 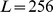. By using periodic boundary conditions and keeping track of unfolded coordinates we avoid forcing the polymers into a confined space.

While simulations of diluted chromosomes can be used to study the effect of looping isolated from the presence of other chains, simulations of polymers in a dense system are necessary to study the formation of chromosome territories and to answer the question whether density-related effects are observable. Thus, it is a natural next step to perform simulations in a system with many chromosomes. For our simulations we choose a linear simulation box of width 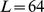 and a density of 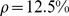, which is similar to the conditions in interphase nuclei. A total of 4096 monomers was thus studied.

Most often, simulational studies map coarse-grained monomers to physical length scales, e.g. by assuming a certain persistence length [31], [38]. Thus, a parameter-dependent comparison between the physical distance of two markers with experimental data from FISH measurements can be conducted. To obtain testable quantitative predictions we follow another, more universal, approach. We derive quantities which are independent on the detailed mapping of the model fiber to the biological chromosome, but can be easily evaluated both from simulational data as well as from experimental data. Such quantities comprise dimensionless higher-order moment ratios of the distance distributions as well as scaling exponents. We show that these quantities do not depend on the chosen level of coarse-graining, i.e. the chain length. Thus, without assuming unknown time and length scales, a sensitive comparison between theory and experiment is possible.

## Results

Modeling chromosomes with complex interaction patterns results in the need to dramatic simplifications in order to allow sufficient relaxation of the fiber within a feasible computational effort. Therefore, we study the looping dynamics for isolated coarse-grained chromatin fibers first. Although such conditions are not found in *in-vivo* experiments, the formation of loops and its influence on the chromosome properties can be studied isolated from density-related effects. In the next step, we present the results of simulations of a system of several chromosomes at biological densities. Since the looping results in confined structures, as will be shown below, density-related effects are only minor and, consequently, the formation of chromosome territories is observed.

### Mean square distance between chromatin segments

We first show that the Dynamic Loop model is in agreement with experimental data from FISH measurements [12], [40], which provide information about the relative physical distance between two target sites. The mean squared distance value 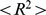 between those target sites in relation to genomic distance 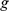 between them can be compared to polymer models. The random walk (RW) and self-avoiding walk (SAW) polymer models predict this mean squared distance to increase monotonically with the distance between two FISH markers,
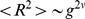
(1)where 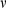 is a model-dependent parameter [41]. In principle, such a scaling is only valid for the end-to-end distances, however, we want to stress that in the absence of other interactions, equation (1) is approximately valid for genomic separations 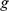 of interest much larger than the persistence length 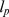 of the chromatin fiber.

The confined space of the nucleus renders the random walk and self-avoiding walk polymer model inadequate. A 100 Mb chromosome with assumed Kuhn length of approximately 300 nm [8] in a 30 nm fiber packing (300 nm 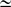 30 kb) would extend on average to 17.3 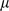
m in the random walk case, whereas the average diameter of a nucleus is of the order of 10 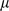
m. The globular state model fails for other reasons [32]. Recent experiments [12], however, clearly revealed that while the mean square distance increases monotonically with genomic separation on short distances up to a few Mb, a leveling-off is observed for larger genomic separations. This confined folding is observed on a scale of about 2 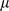
m, far below the diameter of the nucleus but consistent with the estimated size of chromosome territories [7]. The random loop model [12], [42] explains the behavior by the formation of random loops, without invoking a confined geometry a priori.

**Figure 1:**
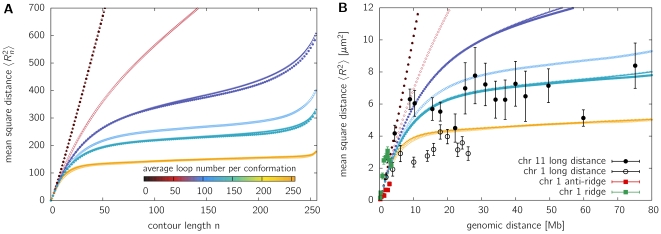
Mean square distance 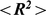 in relation to contour length for an isolated fiber. **A**. The mean square distance 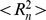 between two beads separated by contour length 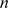. Isolated polymers of length 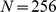 have been fully equilibrated for various looping probabilities 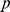. These probabilities are plotted with different colors depending on the resulting average number of loops per conformation. Simulations have been performed using various lifetimes of loops, the results are displayed by different symbols (triangles [▴] for 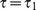, open diamonds [◊] for 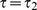 and filled circles [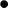] for 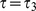). The mean square distance displays a leveling off for average loop numbers beginning at a number of 80. **B**. Comparison of the mean square distance to experimental data taken from Mateos-Langerak et al. [12]. Measurements have been performed on the q-arm of human interphase chromosome 1 and 11. Each bead of the model fiber represents a 400 kb stretch of chromatin with an average extension of 480 nm.

We first considered the mean square distance between two beads in the DL model for isolated chains. Given a chain of length 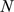 with monomer positions denoted by 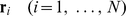, the average is calculated over a set 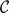 of independent conformations as well as over different reference points inside the chain
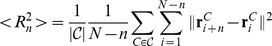
(2)
Fig. 1 shows the results of the model for a chain of length 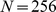. The looping probability 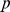 is varied such that different values for the average number of loops are obtained. Lacking knowledge of the biological lifetime of the loops, results are shown for three different values of 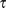 depending on the relaxation time of the chromosomes 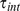 (triangles [▴] for 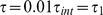, open diamonds [◊] for 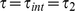 and filled circles [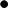] for 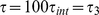, see Materials&Methods). The model displays a cross-over from self-avoiding walk behavior (small number of loops) to a leveling-off in the mean square distance. Such a plateau level is recovered if the average number of loops on a coarse-grained chromosome is larger than about 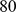. This result is independent of the lifetime of the loops as long as the average number of loops remains the same, indicating that the lifetime has no direct influence on the statistical equilibrium properties. These findings clearly show that no long-range interactions are necessary for forcing the polymer to collapse but a purely diffusional motion together with chromatin-chromatin binding affinity suffices to achieve this.

To quantitatively compare the model to experimental data, we assume each bead to represent a 400 kb segment of chromatin with an average extension of 480 nm (in agreement with experimental data [12]). To ensure that the qualitative results are not dependent on chain length, we studied the mean square distance for 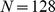 and 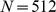 (see Figure S1). In all three cases a leveling-off is observed, indicating that the observed results are independent on scale and the applied coarse-graining is justified.

### Self-organized formation of large loops

**Figure 2:**
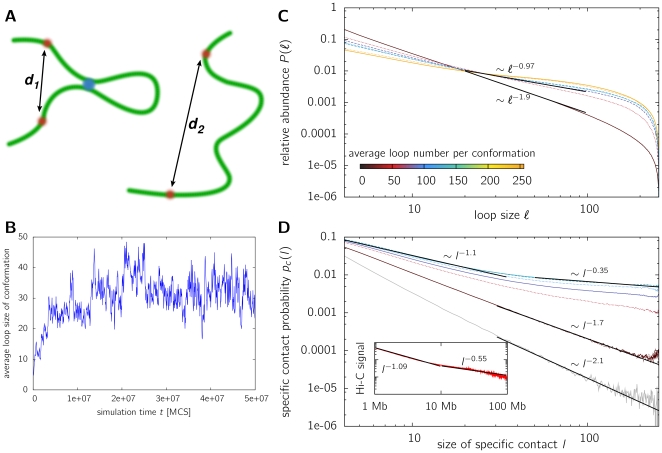
Size distribution of loops and random contacts. **A**. A sketch displaying the facilitated formation of large-loops under the existence of small loops. For a linear polymer, the probability that two chromatin segments marked by red dots co-located by random diffusion is small (right image). Once a small loop has formed in the model (blue dot, left image), the co-localization of the red markers becomes much more frequent. The reason is that the formation of a loop decreases the average distance 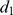 between the red markers compared to the linear case *d*_2_. **B**. This figure displays the average loop size of a conformation during the run. Starting from an equilibrated self-avoiding walk conformation (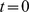), small loops form by random collisions. This enhances the probability of segments further apart to come into contact, thus the average loop-size increases, allowing even loop-sizes of the length of the chain. **C**. Shown is the size distribution of functional chromatin loops of model polymers with 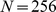 beads. Model polymers were fully equilibrated and the loop size distribution was determined for various looping probabilities 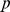 (for reasons of comparison the average number of loops per conformation is displayed by a color code) and lifetimes 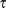 of the functional loops (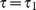 solid line, 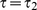 dotted line, 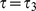 dashed line). Looping lifetimes are chosen relative to the relaxation time, see eq. 6. In an intermediate region, away from the chain ends, the curve can be roughly fitted to a power-law 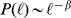. Increasing the loop number results in a markedly smaller exponent, leading to a high probability for large loops. **D**. The contact probability 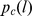 for two specific sites with genomic separation (contour length) 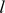 to become co-localized. Shown are the results for equilibrated model polymers with 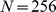 beads and various looping probabilities 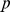. The contact probability decreases as a power-law 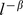 with genomic separation for separations 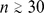, the exponent strongly depending on looping probability. The grey line represents the self-avoiding walk. Again, the co-localization probability is strongly increasing due to diffusion-based looping. The inset shows recent Hi-C data [45] (average signal vs. loop length). Similar to the model, the data shows two regimes with different exponents.

Loop formation is a central process for the transcriptional regulation in higher eukaryotes. Several studies indicated that co-localization of chromatin segments results in activation or repression of genes [20]. Hypotheses of loop formation range from the attachment to a structure called nuclear matrix [43] to the formation of transcription factories [24], in which transcriptionally active genes come together, forcing the intervening DNA to loop out. It has been proposed that rossette-like loops arise in a self-organized manner due to the heterogeneity of the fiber [44]. Recently it has been shown [28], [29] that loops can promote territory formation with a simple model using fixed loops. However, such a kind of looping does not yield a correct description for the relative positioning of two markers [12]. Rather, it has been shown by 4C experiments [25], that loops exist on scales up to several Mb. 3C/4C/5C and the newly developed Hi-C [45] techniques provide an experimental method to measure loop probabilities and distributions. Therefore, we next investigated how the model alters the distribution and frequency of genes to become co-located. Again, we favor measures that do not depend on the level of coarse-graining and parameters like persistence length. One such measure is the decay of the contact probability and abundance with genomic separation 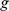. Consider a random walk polymer chain. Clearly, the probability that two beads 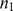 and 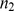 come into contact decreases with the separation 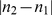. More precisely, we obtain a power-law behavior [35]

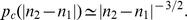
(3)Consider two genes separated by 10 Mb. Assuming a Kuhn segment length of 300 nm [38] consisting of 30 kb chromatin, the probability of co-localization is in the order of 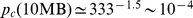. How, then, does the cell nucleus manage to co-locate different chromatin segments in a reasonable time? To answer this question, we look at the formation of functional loops in our model and its size distribution 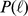. Interestingly, the diffusional pathway to loop formation results in a size distribution of functional loops 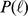 which is quite different from the small random contact probabilities of a RW or SAW model (Fig. 2C). Strikingly the probability of having a loop in the size-range of the chain length is enhanced by over two orders of magnitude. The increase in probability for large-scale loops in contrast to small-scale loops can be explained on an intuitive basis: Starting from a linear chain, the diffusional process will bring monomers close together which are not so far away along the contour of the chain. Loop formation will be dominated by small-sized loops as equation (3) still holds. However, as more and more small loops form, even parts of the polymer located further apart come closer together (Fig. 2A), thus enhancing the probability of contact. Figure 2B visualizes for one simulation run the average loop size along the simulation time. We find that this average loop size increases fast and then fluctuates around an equilibrium value. Therefore diffusional looping seems to be a quite fast and effective method of large loop formation.

To allow a comparison to experimental data from 4C and 5C experiments we determine two measures. Firstly, the size-distribution 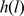 of random contacts (as 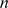 C experiments do not only measure functional contacts) between two chromatin segments. Secondly, the specific contact probability 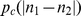 that two segments at position 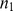 and 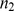 are in contact. From eq. (3) we know that for a random walk the specific contact probability has a power-law behavior depending on the length 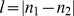 given by 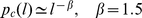. A power-law behavior is also found for the self-avoiding walk, where the exponent is determined in Fig. 2D to 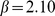. In fact, scaling theory predicts [35] for self-avoiding or random walk polymers that the contact probability of the end-points of a polymer scales as 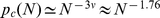. Our analysis suggests that the contact probability for intra-chain segments decreases more strongly. This is somehow expected, as intra-chain segments have less entropic degrees of freedom and are surrounded by a higher density of adjacent beads than the end points, making contacts with beads further away less likely. Our polymer model as well displays a power-law behavior of the co-localization probability 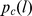 (Fig. 2D). However, two different regimes have to be distinguished. For genomic separations 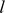 in the size range of the whole chromosome a different exponent is found as in the size range below about 15% of the fiber length. In the regime of probability-values 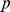 where leveling-off in the mean square distance is observed, we find exponents of about 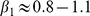 for smaller genomic separations in the order of 10 Mb and 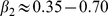 for large genomic separations in the order of 100 Mb (table 1). Intriguingly, the probability of specific contacts between far-apart chromatin segments is increased by over two orders of magnitude compared to the self-avoiding walk. Increasing the looping probability and thus the average number of loops per chain results in smaller exponents 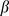. Interestingly, this result is in close agreement with recent results from Hi-C data [45] where an exponent of 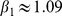 has been observed in a region between 500 kb and 7 Mb (see inset of Figure 2D). For genomic separations above 10 Mb we find a scaling exponent of 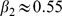, consistent with the smaller scaling exponent found in our model. Similar results are found for other chain lengths (Figure S2 and Figure S3).

**Table 1:** Decay exponents of the random contact probabilities with genomic separation for direct comparison to 4C and 5C experiments.

number of loops	loop lifetime 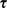	symbol	exponent 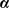	exponent 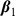	exponent 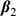
19.0	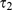	dark-red ⋄	2.01	1.78	1.66
19.0	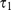	dark-red ▴	2.00	1.76	1.68
19.1	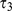	dark-red 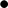	2.05	1.79	1.64
59.2	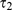	red ⋄	1.19	1.24	0.70
87.0	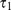	blue ▴	0.95	1.09	0.43
87.2	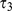	blue 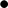	0.92	1.11	0.38
112	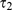	light-blue ⋄	0.84	1.00	0.30
131	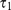	light-blue ▴	0.81	0.94	0.35
247	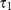	yellow ▴	0.70	0.79	0.35

Shown are the resulting exponents 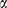 of a power-law fit to the size distribution of random contacts 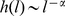. A fit to the specific contact probability 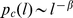 has been performed both in the region of small genomic separations (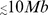) and in the region of genomic separations up to the complete chromosome (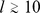 Mb), yielding different exponents 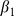 and 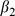 respectively. These exponents can be compared to results from 5C and 4C experiments. Data is displayed for equilibrated chains of length 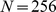 for various looping probabilities 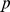, corresponding to different average numbers of loops, and different lifetimes 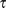 of functional loops. For comparison with Fig. 1, the corresponding symbols are listed.

5C data provides a detailed map of interactions between chromatin segments without a fixed reference point. Thus, it is more natural to look at the relative abundance of contacts 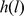 of size 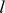, encompassing all fragments of a certain length 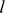 found in the data independent on their position on the genome. A crude power-law fit 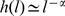 can be conducted here, too (see Figure S4). We find power-law exponents of 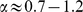 in the range where leveling-off in the mean square distance is observed (cf. Fig. 1). The exponents both for the specific contact probability 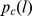 as well as the size-distribution 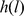 are listed in table 1.

**Figure 3:**
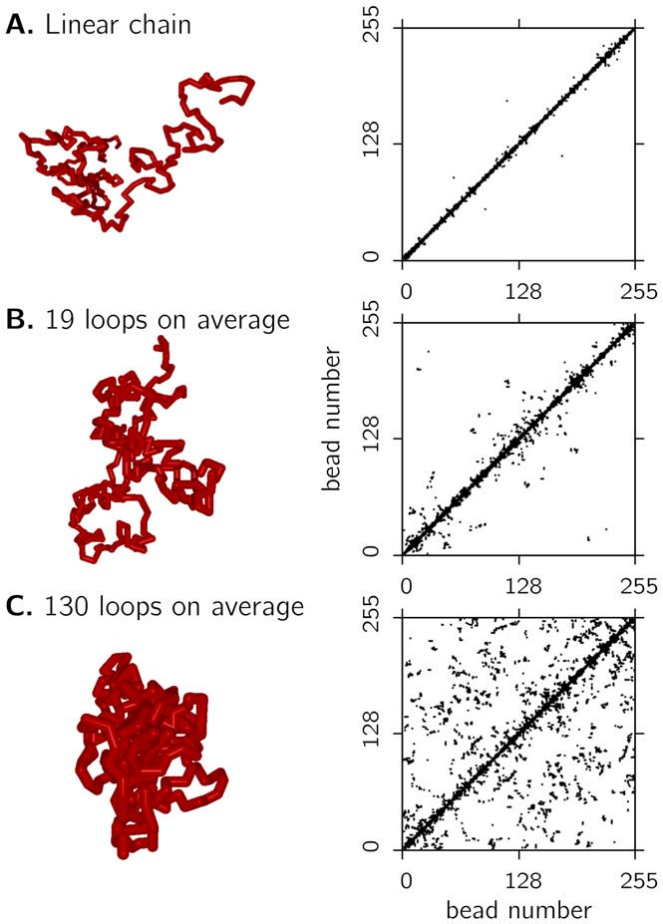
Intra-chromosomal contacts of isolated model polymers. Shown are the results for equilibrated fibers of length 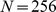 with different looping probabilities. For each parameters set (A. linear chains (no loops), B. on average 19 loops per conformation and C. on average 130 loops per conformation) co-localized beads were determined and marked by a black square. For each image, the contacts of 4 independent polymer conformations are plotted. Linear chains (A) have not so many contacts between beads which are widely separated along the contour of the polymer. Increasing the probability of functional loops (B and C) results in a boost of contacts both between close-by segments as well as between segments having a large genomic separation.

Fig. 3 shows contact maps similar to those obtained by 5C for a 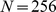 polymer with different looping probabilities. Contacts between any two beads are marked by a black square. For better visibility, in each map contacts of 4 equilibrated conformations are plotted. Clearly, the self-avoiding walk polymer model (Fig. 3A) only has a few contacts between beads located far apart. Increasing the looping probability (Figs 3B and C) results in a strong increase of both the number of loops as well as the abundance of large loops.

Theoretically, the change in the scaling exponent can be explained by the topological changes induced to the fiber on introducing loops. For a polymer network, the looping probability generally behaves like 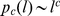 with the exponent 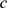 depending on the specific topology, i.e. the vertices, of the network [46]. For more complex networks, i.e. loop configurations, the exponent decreases due to the less conformational degrees of freedom available.

### Cell-to-cell variation and dynamic fluctuations of the distance distribution

While FISH measurements have been used to establish a connection between the mean square distance of two markers and genomic separation [8], [12], [40], a direct comparison to polymer models requires parameters to map one model bead to physical units like nanometers and base pairs. As these parameters are unknown or based on crude estimates [8], [30], it is desirable to introduce dimensionless quantities not dependent on length scale parameters.

For the the random walk (RW), self-avoiding walk (SAW) and the globular state (GS) model, the following higher-order moments of the distance distribution between two markers turned out to be basically independent of genomic separation [32].
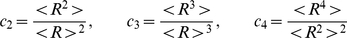
(4)An intrinsic advantage of these measures is that they are dimensionless, i.e. both experiments and models yield a numeric value. Even more important, the ratios carry information about the fluctuations, i.e. the cell-to-cell variation of the measurements.

**Figure 4:**
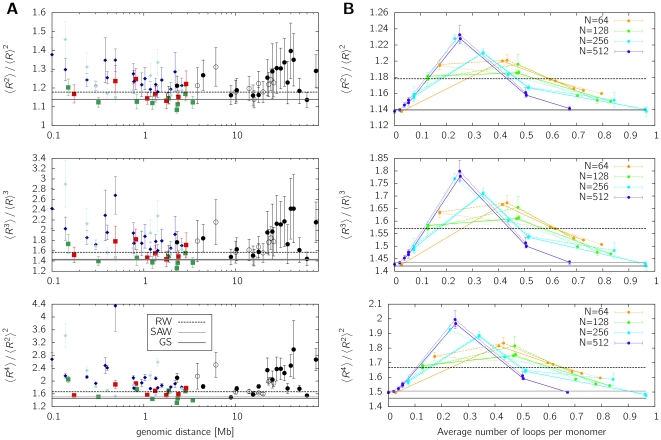
Higher-order moments of the distance distributions for experimental data (A) and for the chromatin model (B) according to **eq. (4)**. **A**. The following experimental data is shown: *Human fibroblasts Chr1* [12]: red ▪ anti-ridge region, green ▪ ridge region, 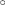 long distance measurements; *Human fibroblast Chr 11* [12]: 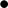 long distance measurements; *Murine Igh locus* [27]: dark blue ⧫ pre-pro-B cells, light blue ⧫ pro-B cells. The data displays strong deviations towards larger fluctuations in comparison to the random walk (RW), self-avoiding walk (SAW) and globular state (GS) polymer model. **B**. Results are shown for simulated polymers of various length (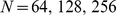 and 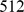) in relation to the average number of loops per monomer, which is related to the looping probability but allows for a better comparison. Although incorporating full excluded volume interactions, fluctuations exceed the random walk value due to probabilistic looping.

One prominent feature of FISH measurements in interphase chromatin is that the fluctuations of the distance distributions are larger than expected from a random walk or self-avoiding walk polymer model [42]. Recently it has been shown that this holds true for the case of compact polymers as well [32], where the fluctuations are even smaller. The ratios given in eq. (4) for experimental data sets from Mateos-Langerak *et al.* [12] as well as Jhunjhunwala et al [27] are presented in Fig. 4A. The figure contains FISH data from human chromosomes 1 and 11 [12], separately measured for ridges (green squares) and anti-ridges (red squares) as well as data from the murine Igh locus [27], which was kindly provided by K. Murre.

The results for the model treated in this paper are shown in Fig. 4B. Model polymers of different length (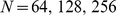 and 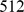) have been equilibrated and averaged over a huge ensemble of conformations encompassing various configurations of loop attachment points. The data is plotted against the average number of loops per monomer to allow for a comparison between different chain lengths. For small looping probabilities, i.e. small average number of loops, the self-avoiding walk behavior is recovered, whereas increasing the looping probability leads to a strong increase in the fluctuations of the system. The higher-order moment ratios markedly exceed the random walk value in the range of loop numbers between 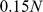 and 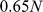. One would expect for a random-walk polymer to have larger fluctuations than a polymer constraint to excluded volume interactions and topological constraints. In our model, the large fluctuations are induced by the dynamic formation of loops, which thus seems to be an important characteristics of chromatin organization. However, it has to be noted that the fluctuations of the model are still too small to explain the moment ratios of most of the experimental data. We will discuss this in more detail in the discussion section.

### The dynamics of looping chromosomes

**Figure 5:**
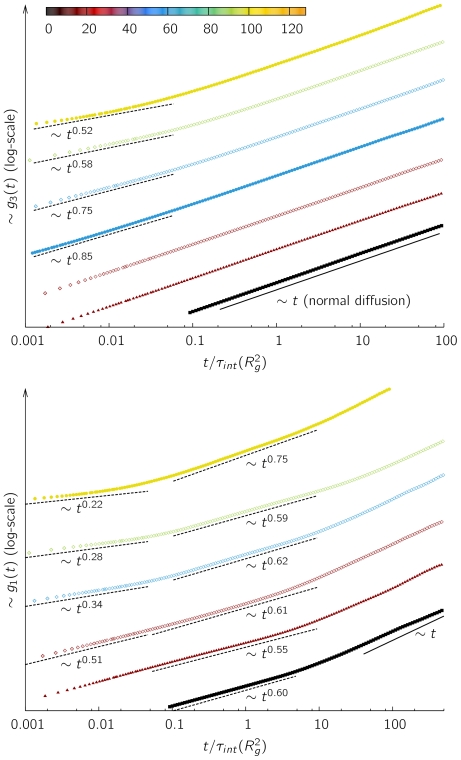
Dynamics of the center of mass and the central monomers. The upper figure shows the motion of the center of mass 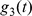 for different parameters for a chain of length 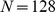. The movement of the polymers' central monomer 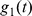 is displayed in the lower figure. The color indicates the average number of loops per chain (see color bar), the point type indicates the loop lifetime (triangles [▴] for 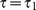, open diamonds [◊] for 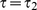 and filled circles [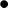] for 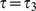). We find subdiffusive behavior with different exponents dependent on looping probability for time scales below the relaxation time of the polymer. For reasons of readability curves are shifted along the y-axis relative to each other.

Finally we study the dynamics of the looping chromatin fibers. The center-of-mass motion of a polymer is measured by 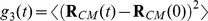. For a self-avoiding walk polymer it shows normal diffusion behavior, i.e. 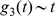. As can be seen in Fig. 5 the chromatin model shows subdiffusive motion 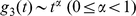 on time scales smaller than the relaxation time of the polymer. The actual diffusion exponent 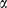 depends on the looping probability 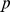. For times larger than the relaxation time one recovers diffusive motion, i.e. 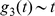, however, this motion is slower than for a normal self-avoiding walk (Figure S5). This is consistent with experimental results showing that chromosome territories do not move significantly [47].

It has to be noted that the regime of large times is not very sensitive for a comparison to experimental data as here the confinement by other polymers comes into play which is not incorporated into the simulations of a single polymer. It is more instructive to look at the motion of the central monomers of a chain on short time scales. The mean square displacement 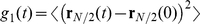 displays a distinct behavior for three different time regimes, which are related to the relaxation time 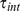 of a chromosome. For 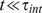 there is a pronounced subdiffusive behavior. The anomalous diffusion exponents range from 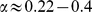 in the regime where leveling-off is observed for the mean square displacement (cf. Fig. 1). For 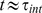 the predictions of classical polymer dynamics become valid again and we find 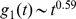 similar to the self-avoiding walk. On large time scales (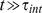), the monomer motion follows the motion of the center of mass, displaying normal Brownian motion with 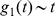.

While at intermediate and large time scales the motion can be described by classical polymer theory, i.e. Rouse dynamics [41], the scaling exponents on the short time scale 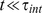 are unexpected. Following the argument in Refs. [37], [38], this time scale is the prevailing one concerning interphase chromosomes. Clearly, such exponents arise due to the constraints induced by looping, which temporarily slows down the motion of chromatin segments at the loop attachment points. Although experimental data is rare, this is consistent with findings of Cabal et al. [17] in yeast. This study showed that the motion of a labelled spot scales like 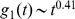 up to 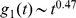. Interestingly they found the exponent to depend on the transcriptional state of the *GAL* genes. This is in support of our conjecture put forward in another publication [12] that the local looping probability may be related to transcriptional activity.

### The formation of aspherical chromosome territories

Polymer theory predicts that equilibrated polymers with a large molecular weight in a semi-dilute solution are strongly intermingling [35]. Various studies, however, indicate that chromosomes occupy discrete functional domains [7], [48], [49]. It was shown above that our model polymers adopt a confined structure by virtue of dynamic looping. Amazingly, this result was obtained without subjecting the system to a confined space (in contrast to Refs. [30], [31]) and without introducing long-range interactions (in contrast the polymer models in Refs. [11], [42], [50]).

Surely, simulating isolated chromatin fibers does not yield complete information about the folding in a dense system as in the nucleus, e.g. the formation of chromosome territories (CTs). To investigate whether probabilistic loops are the reason for the formation of chromosome territories, we set up simulations of chromosomes in a box of width 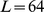 lattice units and a length of 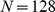. The density of the system 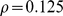 was chosen close to the estimates of chromatin in cell nuclei. Similar values were used in other publications [28].

**Figure 6:**
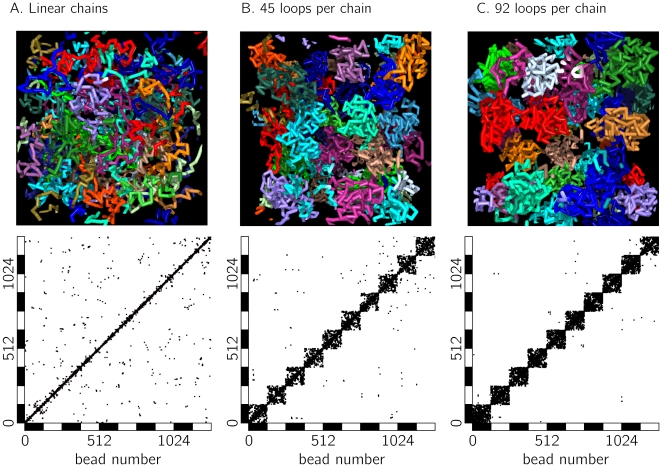
Contact maps and illustrations of chromosomes with different looping probabilities. Simulations were performed in a system with density 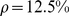 and chains with a coarse-grained length 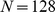. Any contact between two beads is represented by a square in the contact map. Statistics is taken over 5 independent conformations. Not the complete contact map is shown, but only contacts between 10 chains. Linear chains (A) display a lot of intermingling and have abundant contacts with other polymers. The fraction of inter-chromosomal contacts is 15.4%. Increasing the loop-size (B and C) results in more and more confined structures, which are depleted of inter-chromosomal interactions. In (B) chains have on average 45 functional loops (blue ▴ in Fig. 7A), the fraction of inter-chromosomal contacts is reduced to 1.8%. This value decreases even more for chains with an average of 92 loops per conformation (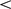
1%, green ▴ in Fig. 7A).

**Figure 7:**
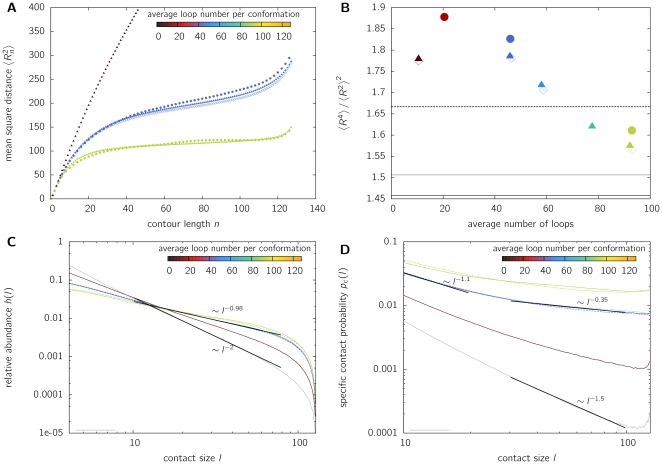
Properties of looping polymers in a dense system. Coarse-grained polymers of length 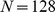 are equilibrated in a system of density 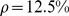. Results are shown for various looping probabilities, which are indicated by the color-coded average number of loops. **A**. Relationship between mean square distance 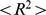 and genomic separation (contour length 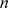). Polymers with small looping probabilities (dark red curve) show a continuous increase of mean distance between two markers with their separation 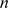. Thus, these polymers do not form discrete territories but intermingle strongly. If the average number of functional loops exceeds 40–50 loops per monomer, a leveling-off is observed and the chromosomes fold into a confined space. **B**. The ratio between higher-order moments 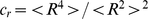 indicates a regime of larger fluctuations than in the random walk case for polymers with 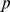-values in the range where leveling-off occurs. The values found are in the size range of 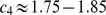. Such large fluctuations, i.e. cell-to-cell variation, are an intrinsic feature of chromatin organization (Fig. 4A), represented in our model by the dynamic formation of probabilistic loops. **C**. The size distribution of random contacts 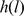 demonstrates that diffusion-based looping facilitates the formation of large loops. Instead of decreasing with 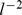 as in the case of linear chains, looping polymers in the parameter range where leveling-off is observed (cf. A) show a power-law behavior of approx. 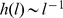. **D**. The probability 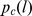 that specific loci on one chromosome co-localize as measured in 4C experiments displays approximately a biphasic power-law behavior. On the scale of the whole chromosome, the contact probability decreases with 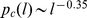, the exact exponent depending on the looping probability. On intermediate length scales a power-law of 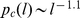 is found in agreement with experimental data [45]. Again, the co-localization probability is greatly enhanced by the formation of functional loops.

An established measure of territory formation is the number of contacts displayed in the contact map [28]. Figure 6 shows such contact maps for different looping probabilities 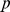. Each map displays contacts between the beads of a subset of 10 model chromosomes out of the system. The beads are numbered consecutively, i.e. bead 0 to 127 belong to chain 1, bead 128 to 256 belong to chain 2 etc. Subsequent chains are alternatingly marked by black and white bars. We find that linear self-avoiding walks (Fig. 6A) without loops display a relatively large number of inter-chromosomal in comparison to intra-chromosomal contacts: 15.4% of the contacts are found to be with other chromosomes. With increasing looping probability 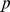, the percentage of contacts between different chromosomes decreases. Fig. 6B displays chains with an average number of 45 loops (blue triangle in Fig. 7A). Here we find that only 1.8% of the contacts are inter-chromosomal and in Fig. 6C (92 loops per chain on average, green triangle in Fig. 7A) this value reduces further to 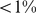. Thus, the level of intermingling between CT's strongly depends on the local looping probabilities. As different local looping probabilities seem to play a dominant role in chromatin organization, this finding could explain different levels of intermingling found in several studies [51], [52]. Branco et al. [51] found out that about 20% of the chromosomes are in contact with other chromosomes. To be able to compare these experiments with our model, we determined the fraction of monomers of one chromosome which are close to neighbouring chromosomes. For linear chains without loops (Figure 6A), we find an overlap fraction of about 50%, i.e. the polymers intermingle strongly. For chromosomes with 45 loops per chain on average (Figure 6B), the overlap fraction is about 15%, i.e. slightly smaller than the average observed for chromosomes. Branco et al. indeed observed different overlap fractions dependent on chromosome activity, thus different local levels of looping might indeed mediate such different overlap fractions.

Thus, a disentangling of the fibers, which has been estimated to require a huge amount of time or the action of topoisomerase II [37] is not necessary. Rather, loop formation alone induces a strong repulsive interaction between different chromosomes; a finding which has been quantified for ring polymers [53] and rosette structures [54]. In mitosis, chromatin adopts a compact state, where different chromosomes are unentangled and well-separated. At the onset of interphase, the loop formation forces the chromosomes to a more open, but confined structure, which results in the formation of CTs without requiring the assumption of unequilibrated polymers [38].

We find that the predictions from the study of isolated model chromosomes are still valid for a dense system of chromatin. Amongst others, this is a direct consequence of loop-based segregation observed in Fig. 6. Fig. 7A shows the mean square distance between two model beads in relation to contour length 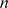 (in biological terms: genomic distance 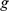). Similar to the results of Fig. 1, the mean square distance displays a leveling-off for average loop numbers larger than about 45 loops per coarse-grained monomer. Obviously, for small looping probabilities 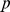 (red curve) or self-avoiding walks (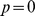, not shown), polymers do not level off, thus they do not form separate territories. The behavior of territory formation and segregation is a distinct result of loop formation.

While the mean square distance 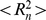 displays a leveling-off for several polymer models (e.g. globular state [31], [32], random walk in a confined space [30], etc.), a more sensitive measure are again the dimensionless ratios of higher order moments given by eq. (4). As the fluctuation regime could possibly change under the transition from isolated polymers to a dense system, we investigate the ratio 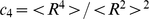. Fig. 7B shows that fluctuations are larger than predicted by the random walk, self-avoiding walk or globular state model. In the regime where a leveling-off is obtained in Fig. 7A, i.e. the average loop number is larger than about 45, the moment ratios are approximately in the range 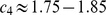.

The relative abundance of contacts 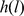 is displayed in Fig. 7C for polymers in a dense system. Again, the co-localization frequency is greatly enhanced by the formation of functional loops. A crude power-law fit 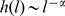 results in exponents of 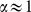 and smaller in the region where a leveling-off is observed in the mean square distance. Similar results are found for the specific contact probability 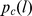 (Fig. 7C), which display a biphasic behaviour already observed in the case of isolated chromosomes (Fig. 2). In the size range of large genomic separations in the order of the entire chromosome, the contact probability decreases with a power-law 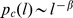 with exponents starting from 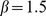 in the self-avoiding walk model to 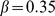 in the parameter range where leveling-off is observed. On intermediate scales (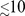 Mb), for biologically relevant looping probabilities, an exponent of 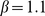 is found. Amazingly, a similar value of 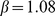 has been recently found by Hi-C experiments [45] on a scale between 500 kb to 7 Mb.

Isolated model chromosomes displayed a pronounced conformational asphericity (Figure S6). A similar behavior is also found for chromosomes in a dense system. In fact, deviations from a sphere-like shape are expected for the self-avoiding walk as well as the random walk model [55], however, not for a compact globular state polymer [32]. Whereas looping polymers can adopt a highly compacted state, their properties differ clearly from a globular state. Indeed, the shape of simulated chromosomes territories is not spherical as one would expect for compact polymers, rather we find that the gyration ellipsoid has a prolate shape. The ratios of the gyration tensor's eigenvalues are listed in table 2. In the parameter range where the mean square distance displays a leveling off, we find ratios of the eigenvalues 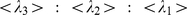 in the regime between 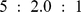 and 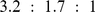. These values are smaller than those of Rosa *et al.* [38] for ring polymers and consistent with those of looping polymers [28]. A non-spherical shape of CTs has also been found in experimental studies [56], [57]. Mouse chromosomes exhibit an aspherical shape approximated by ellipsoids with axis ratios 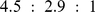. A one-to-one correspondence of these numbers with results from the shape of the gyration ellipsoid, however, can not be established.

**Table 2:** Shape parameters of simulated chromosomes.

number of loops	loop lifetime	symbol	eigenvalue ratios	axis ratios
10.5	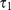	red ▴	10.2 ∶ 2.6 ∶ 1	3.2 ∶ 1.6 ∶ 1
45.9	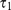	blue ▴	4.9 ∶ 2.0 ∶ 1	2.2 ∶ 1.4 ∶ 1
45.9	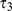	blue 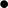	5.0 ∶ 2.0 ∶ 1	2.2 ∶ 1.4 ∶ 1
92.1	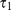	green ▴	3.3 ∶ 1.7 ∶ 1	1.8 ∶ 1.3 ∶ 1
93.0	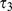	green 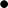	3.2 ∶ 1.7 ∶ 1	1.8 ∶ 1.3 ∶ 1

Shown are the results for equilibrated coarse-grained polymers of length 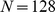 in a melt of density 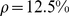. Results have been calculated using various looping probabilities and lifetimes of the loops. Correspondence to Fig. 7A is established via the symbol, which is shown in the third column. The shape is parameterized by the ratios of the eigenvalues of the gyration ellipsoid, corresponding to the squares of its axis lengths. The axis ratios 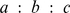 are listed for comparison with other studies (e.g. Cook et al. [28]).

## Discussion

In this study, a polymer model was presented where loops form dynamically on the basis of diffusional collisions. We use Monte Carlo simulations to demonstrate the effect of such a kind of loop formation. While loops have been recognized as an ubiquitous feature in transcriptional regulation, the pathways of its formation remained unclear and most polymer models proposing loops so far did not explain the transport mechanisms by which two parts of chromatin become co-located. Our results suggest that even large loops can arise without active transport mechanisms. Our model neither assumes a confined geometry nor any long-range interactions. Loop formation is based on the diffusional motion of the fiber. Collisions lead to a probabilistic chromatin-chromatin interaction which forces the participating regions to be co-located for a certain time. The probabilistic nature of the interactions is meant to mimic the effect of chromatin binding factors on chromatin-chromatin interactions. Although this Dynamic Loop model is kept minimal, it reproduces many experimental results quantitatively, highlighting the possibility that chromatin folding is tightly related to function through the loop formation process.

One of our major results is that dynamic loop formation drives chromosomes into an entropically segregated state. Indeed, linear polymers intermingle freely (Fig. 6A) in agreement with polymer theory [35]. Looping polymers, in contrast, fold into a confined space (Fig. 1). Such a confinement is also observed for the globular state polymer model, which, however, displays a markedly different fluctuation regime than the experimental data [32]. The importance of looping on the formation of chromosome territories has been investigated recently by Cook *et al.* [28]. In their qualitative study, rosette-structures with fixed loop attachment points are used. While this model can be used as a simple model for studying entropic effects of looping, it does not explain fluctuations in FISH data [12], [42].

The second important result of this study concerns the pathway of the formation of large loops. Results from 3C/4C/5C experiments reveal that loops are abundant on the short scale [58]. Nevertheless, functional loops on the scale of several mega basepairs have been detected in 4C experiments [25]. While the probability of specific random contacts 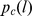 decreases strongly with site separation 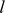 for linear polymers (given by a power-law behavior 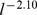), the contact probability is increased by over two orders of magnitude when introducing loops (Fig. 2). Obviously, small functional loops which can easily co-localize by diffusional motion strongly support the formation of long-distance contacts. The contact distribution 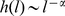 displays power-law exponents of 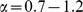, the contact probability 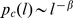 exponents in the size range between 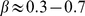. This is in agreement with recent experimental data by Lieberman-Aiden *et al.* [45], however, their interpretation in terms of a fractional globule differs from ours. Clearly, a fractional globule, where the physical distance between two loci scales with 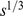 [59], is in contrast to experimental findings from FISH data [12].

The impossibility to perform Monte-Carlo simulations on a very detailed scale requires a coarse-graining procedure. Looking at large-scale features above the persistence length 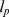, such an approach is well-justified [35]. For linear polymers, scaling laws provide a simple way of rescaling a polymer. For a model with loops, the connection between chain length, bond length and looping probability 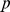 is non-trivial. In fact, even for chromatin models using linear chains (see Refs. [30], [31], [38]), the establishment of a correspondence between simulational units and biological units requires the knowledge of the persistence length of chromatin. The latter has been estimated by fitting a random walk model [30] or a worm-like chain model [8] to FISH data. Estimates on the persistence length range from 40–220 nm [36]. For a quantitative comparison to experimental data we derived measures independent on both the level of coarsening and unknown biological parameters. These can be easily evaluated both for experimental data as well as polymer models. Amongst others, these measures comprise the power-law exponent of the contact distribution (Fig. 2 and table 1), the dimensionless higher-order moment ratios of the distance distribution between two FISH markers (Fig. 4), the asphericity of chromosomes and finally the diffusion exponents (Fig. 5).

The DL model studied here displays a pronounced aspherical elongated shape (table 2) which is also found in experiments [6]. Consistent with experimental data in yeast, the motion of single monomers is subdiffusive (Fig. 5); the actual subdiffusion exponent depends on the looping probability, which was suggested to be closely related to transcriptional activity [12]. A good agreement with experimental data is obtained for the higher-order moments of the distance distribution. Surprisingly, the moment ratios (given in eq. (4)), which display fluctuations of the distance distributions, exceed the random walk value for looping probabilities 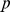 in the range where a leveling off in the mean square distance is observed. This is not necessarily expected for a model with excluded volume which restricts the degrees of freedom and therefore shows less fluctuations. The increase of the fluctuations with respect to the self-avoiding walk is due to the dynamic formation of loops. However, several independent experiments [12], [27] consistently show even larger fluctuations. We suspect two major reasons for this: First, the chromatin fiber is not a homogeneous polymer and there is evidence that looping probabilities vary depending on the transcriptional state [12]. Secondly, inside the nucleus, topoisomerase-II might effectively counteract excluded volume interactions, resulting in an underestimate of the fluctuations in our model.

While our model suggests that chromosome segregation might be driven by the diffusional formation of loops, Rosa and Everaers suggested [38] that segregation is a consequence of large entanglement times. The entanglement times, however, might be strongly reduced by the effect of topoisomerase-II [60]. Notwithstanding that time-scales play an important role, this study reveals that loop formation provides a complementary and fully sufficient mechanism for CT formation.

Importantly, the Dynamic Loop model displays a distinctly different behaviour than the equilibrium or fractal globular state (GS) models, which have been proposed for chromatin organization [31], [45]. First, the GS model does not explain the large fluctuations observed in several experiments (Figure 4), rather it predicts fluctuations being even smaller than for a random walk or self-avoiding walk model. Second, the equilibrium globular state predicts contact probabilities to behave as 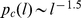 [61], while Hi-C data shows 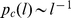, consistent with our model. On the other hand, the fractal globular state predicts the correct contact probabilities but not the leveling-off in the mean square displacement. Third, the globular state predicts rather spherical territories, while our model as well as experimental data shows aspherical chromosome territories. The differences indeed arise by the introduction of probabilistic loops which among others leads to the large fluctuations [42] observed in experiments.

Clearly the chromatin model proposed here does not capture all details of the complex nuclear organization. For reasons of simplicity we neglect the heterogeneity of the chromatin fiber here and assume the same looping probability and chromatin affinity along the complete chromosome. Using such a simple model allowed us to derive the basic properties of chromatin fibers without introducing unnecessarily many parameters. However, experiments clearly show that loop formation is strongly dependent on the differentation state of the cell [25] as well as gene activity [12]. In future work such differences might be incorporated into our model either by adjusting the looping probabilities locally or by distributing specific binding sites along the chromosomes.

## Materials and Methods

The biological model is implemented using Monte Carlo simulations [62]. These Monte Carlo simulations are performed on a lattice in order to simplify the handling of excluded volume. Calculation of excluded volume interactions thereby is reduced to checking whether one lattice site is already occupied or not. Instead of using a simple local-move algorithm on a cubic lattice we employ the bond-fluctuation method introduced by Carmesin [39]. This method has the advantage over other lattice models of allowing 108 different bond vectors; the length of a bond can take the values 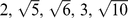 [63]. The bond-fluctuation model is especially suited for dense and compact systems where a lattice algorithm would no longer be feasible due to high rejection rates during the Monte Carlo process. It has successfully been applied to several studies on the static and dynamical properties of polymer systems [63]–[66]. The simulation method fulfills the following important features: (i) it produces unbiased results, i.e. each possible conformation out of the ensemble is sampled with equal probability, (ii) it takes into account excluded volume interactions, i.e. two monomers are not allowed to occupy the same region in space and (iii) using some restrictions on the moves and bond vectors it ensures that no bond crossings can occur during a Monte Carlo step, i.e. it preserves the topological state of the conformation. The algorithm conducts only local moves in order to resemble the dynamics of real polymers [39]. Using a coarse-grained lattice approach is reasonable as we are only interested in features of looping chromatin independent on local structure. Coarse-graining allows us to abstract from the complex environment and highlight the main driving forces and effects of chromatin folding.

Simulations for single polymers are performed on a lattice of size 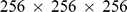. Periodic boundary conditions are used, but the algorithm always keeps track of unfolded coordinates, such that the polymer does not feel any confined volume. The lattice size 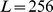 is chosen larger than the radius of gyration 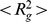 of the chains studied such that effects of the backfolding are negligible.

A dense system of model chromosomes is simulated in a system of size 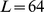 and chain length 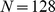. The total number of monomers is 4096, the density 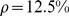.

In order to obtain thermodynamical equilibrated conformations we perform the Metropolis Monte Carlo method. Chromosomes are initially equilibrated as self-avoiding walks using local moves of a monomer to one of the nearest neighbors on the lattice. After the initial equilibration steps, the Monte Carlo algorithm allows for the formation of loops. After each Monte Carlo trial move, one monomer is selected at random. It is then checked whether another monomer on the same chain is in the neighborhood, i.e. co-localized. The co-localization condition is fulfilled whenever the distance between the monomers is less than 3 lattice units. If the two monomers are co-localized, then a loop is formed with a certain probability 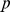. If the loop 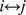 is created, it is assigned a certain lifetime 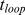 which is drawn from a Poissonian distribution
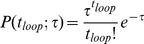
(5)where the parameter 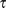 determines the average lifetime of the loops. In the simulations we use three different values of 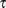:
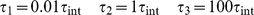
(6)where 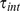 is the integrated autocorrelation time (see below) of the squared radius of gyration for the corresponding self-avoiding walk system. Loop lifetimes are chosen relative to the relaxation time to make results for different values of the other parameters comparable. As an example, we want to give a few numbers here. For a single chain of length 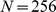, the equilibration time is 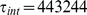 MCS; thus, one corresponding loop lifetime would be 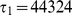 MCS. A looping probability of 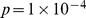 yields an average number of 19 loops per chain, a looping probability of 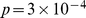 yields an average number of 131 loops per chain.

Since subsequent conformations in the Markov chain created by the Monte Carlo algorithm are highly correlated, one has to perform a certain number of Monte Carlo steps to obtain two independent conformations. For each set of parameters (chain length 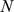, looping probability 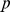 and lifetime of loops 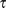) we determine the autocorrelation function 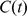 (see e.g. Ref. [67]) of the squared radius of gyration 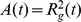


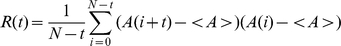


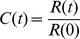
To obtain a reasonable result for the integrated autocorrelation time we have to sample about 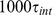 Monte Carlo steps. Then 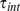 itself is approximated by the following algorithm, which is often called the windowing procedure and was introduced by Sokal [68].

1. Determine 
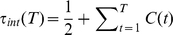
.
2. Choose 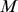 to be the smallest integer such that 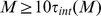. Then 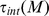 is supposed to be the best approximation of the autocorrelation time.

Another criterion to ensure the uncorrelatedness of subsequent conformations is given by the motion of the center of mass. This method has been used for example by Mueller *et al.* in his study on ring polymers [65]. Here we determine the function
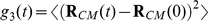
The time of interest, 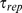, after which the center of mass has moved at about one radius of gyration, is defined by
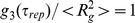
(7)We consider two subsequent conformations as uncorrelated after 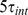 Monte Carlo steps. Actually, for each set of parameters considered here, we found that after this time the center of mass has moved on average at least by one radius of gyration, i.e. 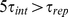.

Simulations of even small polymers are very time-consuming due to the looping interaction and the resulting compactness of the polymers. Furthermore, simulation runs have to be quite long to capture the dynamics of loop formation. We have used the Helics2- and bwGrid parallel computing facilities at the Interdisciplinary Center for Scientific Research (IWR) at the University of Heidelberg. For each set of parameters 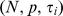 we created 10 000–100 000 independent conformations. We study polymers of lengths 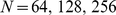 and 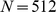. The looping probabilities are chosen such that the average number of loops in the resulting conformational ensemble is between zero and 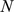. The lifetimes of the loops are chosen from the set given in eq. (6).

## Supporting Information

Figure S1 Mean square distance for various N.
(0.19 MB PDF)

Figure S2 Loop size distribution and specific contact probabilities for N  =  128.
(0.11 MB PDF)

Figure S3 Loop size distribution and specific contact probabilities for N  =  512.
(0.12 MB PDF)

Figure S4 Relative abundance of contacts h(l) in relation to genomic separation l.
(0.15 MB PDF)

Figure S5 Dynamics of the center of mass and motion of the central monomers.
(0.12 MB PDF)

Figure S6 The shape of chromosomes.
(1.41 MB PDF)
