# Supplementary material for: Diffusion-Driven Looping Provides a Consistent Framework for Chromatin Organization
Source: PLoS One. 2010 Aug 25;5(8):e12218. doi: 10.1371/journal.pone.0012218 (PMC2928267; doi:10.1371/journal.pone.0012218)

Supplementary Figure 1

Mean square distance $\langle R_n^2 \rangle$ in relation to contour length for an isolated fiber. Both figures show the mean square distance between two beads separated by contour length n. In the upper-most figure, results for a chain length of $N = 128$ are shown. The bottom-most figure shows results for $N = 512$. Isolated polymers have been fully equilibrated for various looping probabilities p. The p-values are plotted with different colors depending on the resulting average number of loops per conformation. Simulations have been performed using various lifetimes of loops, which are chosen relative to the relaxation time τ_{int} of the polymer. The results are displayed by different symbols (triangles [▲] for $\tau = \tau_1 = 0.01\tau_{int}$, open diamonds [◊] for $\tau = \tau_2 = \tau_{int}$ and filled circles [•] for $\tau = \tau_3 = 100\tau_{int}$). The mean square distance displays a leveling-off for all chain lengths studied, inducing a confined folding of chromosomes based on dynamic looping.

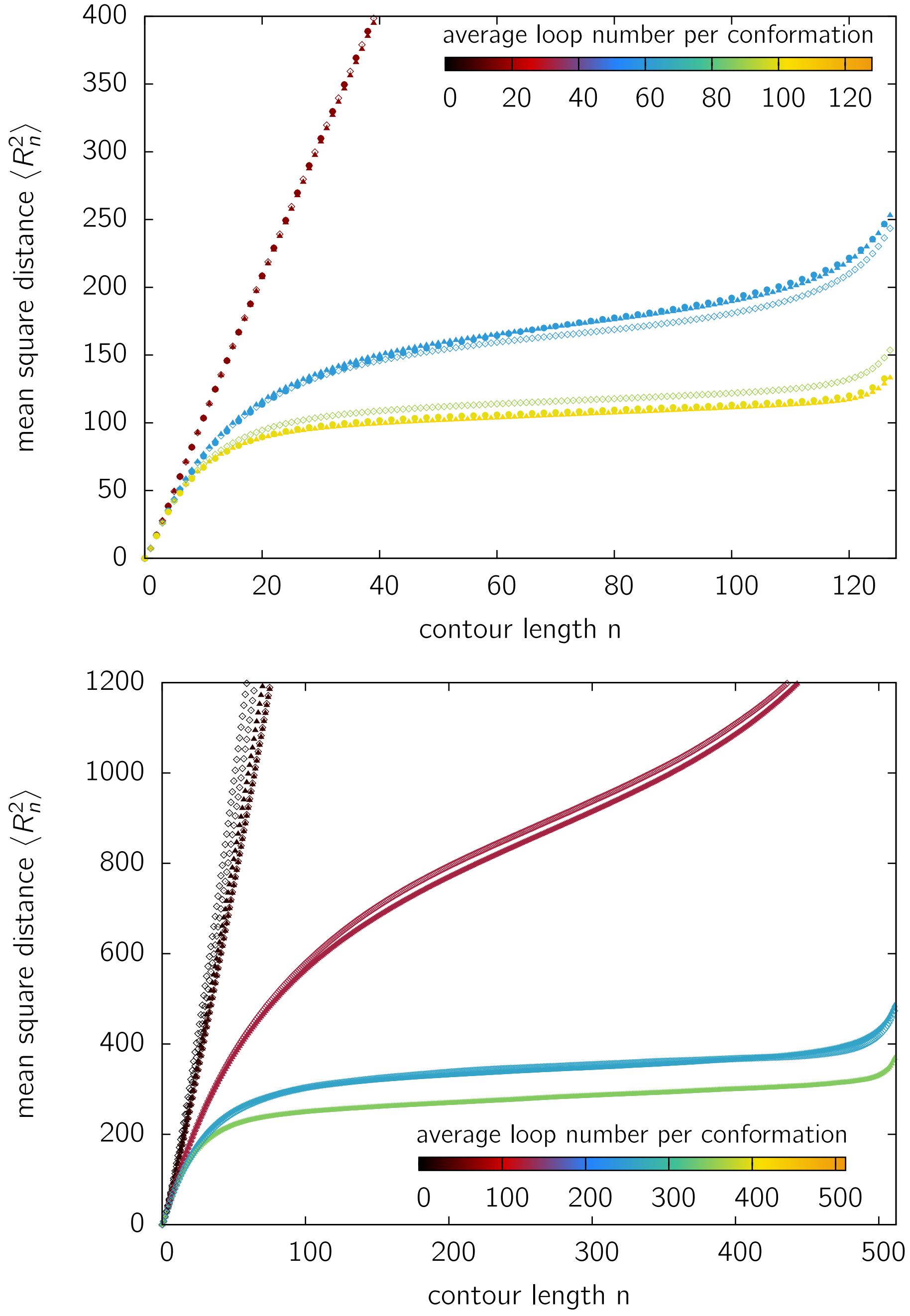

Supplement: Figure S1 — Mean square distance for various N. (0.19 MB PDF) [file pone.0012218.s001.pdf]
