# Supplementary material for: Diffusion-Driven Looping Provides a Consistent Framework for Chromatin Organization
Source: PLoS One. 2010 Aug 25;5(8):e12218. doi: 10.1371/journal.pone.0012218 (PMC2928267; doi:10.1371/journal.pone.0012218)

Supplementary Figure 2

Loop size distribution and specific contact probabilities for $N = 128$. Upper-most figure: Shown is the size distribution $P(\ell)$ of functional chromatin loops of model polymers with $N = 128$ beads. Model polymers were fully equilibrated and the loop size distribution was determined for various looping probabilities p (for reasons of comparison the average number of loops per conformation is displayed by a color code) and lifetimes τ of the functional loops. Looping lifetimes are chosen relative to the relaxation time (cf. Materials & Methods in the manuscript). Increasing the loop number results in a markedly smaller exponent, leading to a high probability for large loops. **Bottom-most figure:** The contact probability $p_c(l)$ for two specific sites with genomic separation (contour length) l to be co-localized. Shown are the results for equilibrated model polymers with $N = 128$ beads and various looping probabilities p. The contact probability decreases as a power-law $l^{-\beta}$ with a biphasic behaviour, the exponent changing at about $l \approx 15\%$ of the chromosome length. The grey line represents the self-avoiding walk. Again, the co-localization probability is strongly increasing due to diffusion-based looping.

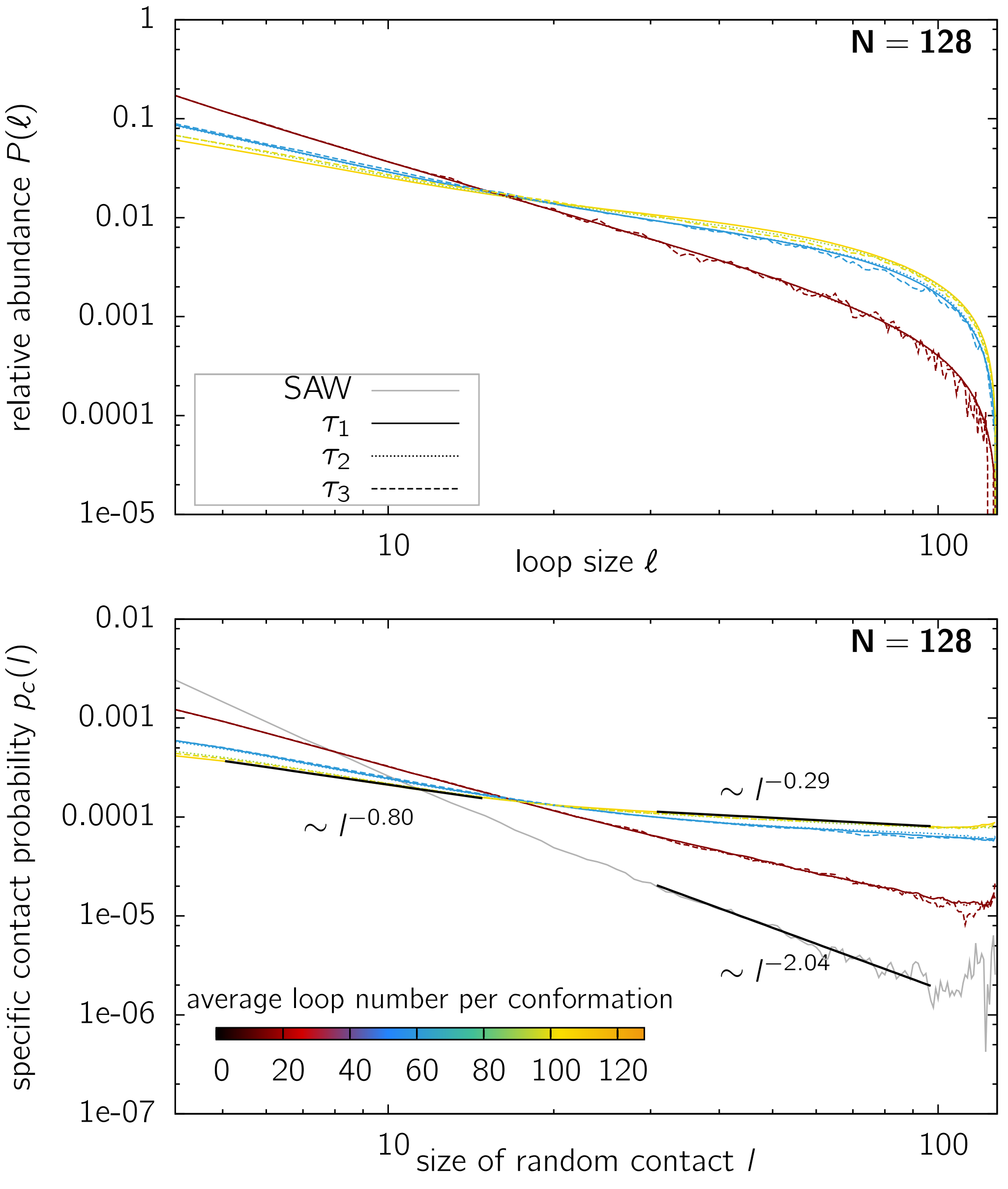

Supplement: Figure S2 — Loop size distribution and specific contact probabilities for N = 128. (0.11 MB PDF) [file pone.0012218.s002.pdf]
