# Supplementary material for: Diffusion-Driven Looping Provides a Consistent Framework for Chromatin Organization
Source: PLoS One. 2010 Aug 25;5(8):e12218. doi: 10.1371/journal.pone.0012218 (PMC2928267; doi:10.1371/journal.pone.0012218)

Supplementary Figure 4

Relative abundance of contacts $h(l)$ in relation to genomic separation l. The size distribution of random contacts $h(l)$ is shown for chains of length $N = 256$. Simulations for different looping probabilities are indicated by a color code. A crude power-law fit $h(l) \sim l^{-\alpha}$ has been performed to the data, showing that the exponent decreases with increasing looping probability.

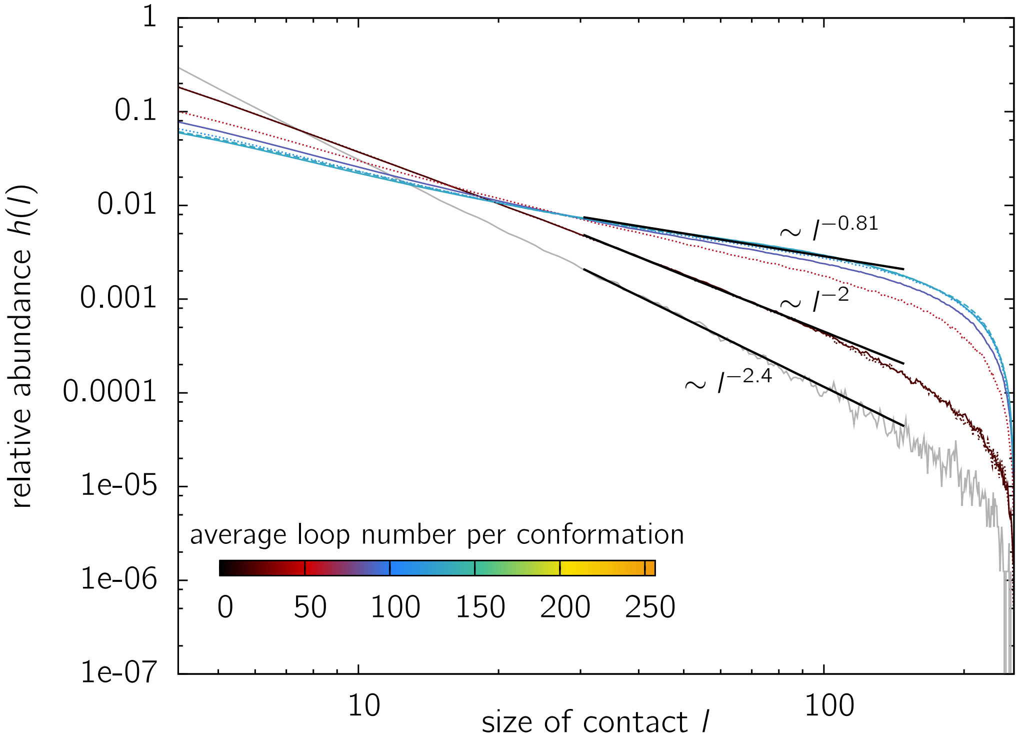

Supplement: Figure S4 — Relative abundance of contacts h(l) in relation to genomic separation l. (0.15 MB PDF) [file pone.0012218.s004.pdf]
