# Supplementary material for: Diffusion-Driven Looping Provides a Consistent Framework for Chromatin Organization
Source: PLoS One. 2010 Aug 25;5(8):e12218. doi: 10.1371/journal.pone.0012218 (PMC2928267; doi:10.1371/journal.pone.0012218)

Supplementary Figure 5

Dynamics of the center of mass and motion of the central monomers. The upper figure shows the motion of the center of mass $g_3(t)$ using a chain length of $N = 128$. The data is shown for different values of the looping probability p, indicated by a color-code for the average number of loops. The lifetime of loops is indicated by the symbol (triangles [▲] for $\tau = \tau_1$, open diamonds [◊] for $\tau = \tau_2$ and filled circles [•] for $\tau = \tau_3$). The movement of the polymers' central monomer $g_1(t)$ is displayed in the lower figure. The unscaled data shows that introducing loops in the system slows down the motion of the polymer as well as of single monomers.

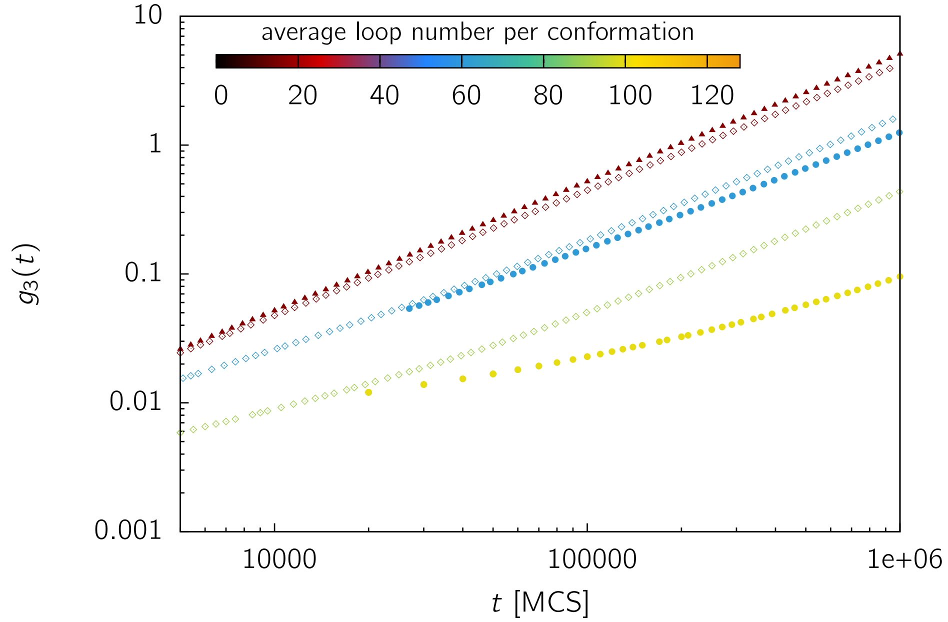

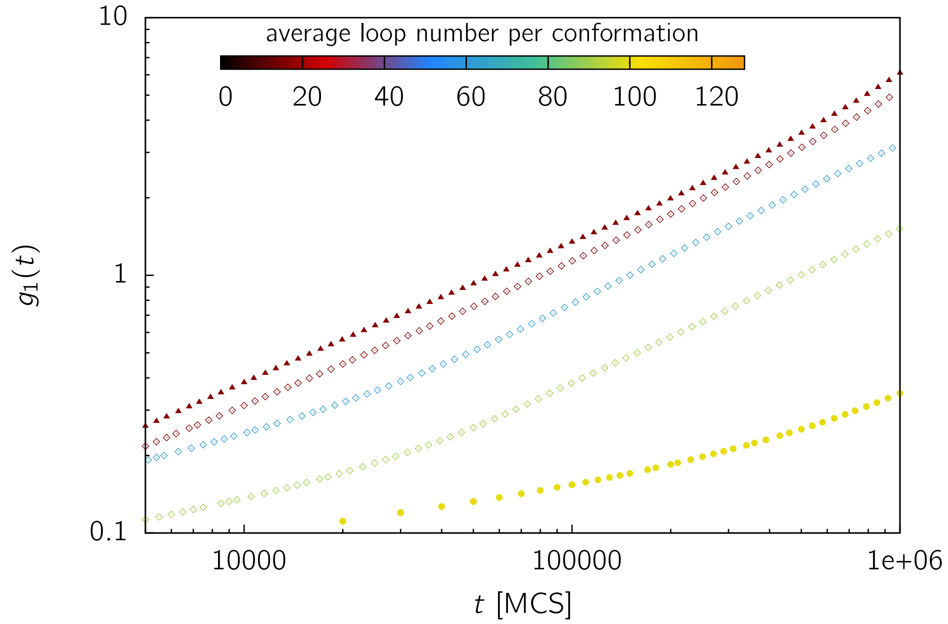

Supplement: Figure S5 — Dynamics of the center of mass and motion of the central monomers. (0.12 MB PDF) [file pone.0012218.s005.pdf]
