# Supplementary material for: Diffusion-Driven Looping Provides a Consistent Framework for Chromatin Organization
Source: PLoS One. 2010 Aug 25;5(8):e12218. doi: 10.1371/journal.pone.0012218 (PMC2928267; doi:10.1371/journal.pone.0012218)
Supplement: Figure S6 — The shape of chromosomes. (1.41 MB PDF) [file pone.0012218.s006.pdf]

Supplementary Figure 6 - The shape of chromosomes

Flourescent labeling of whole chromosomes [1–4] or extended regions of chromosomes [5] can be used to determine the shape of chromosomes. Goetze et al. [5] quantitatively found that chromosomal regions show pronounced deviations from a spherical shape and are correlated to transcriptional activity. Using a wavelet method, Khalil et al. [6] found that the shape of a chromosome territory in mouse is highly nonspherical and can be best approximated by an ellipsoid with average axis length ratios of 4.5 : 2.9 : 1. However, strong differences in shape and positioning were found depending on cell type [7–9] and chromosome state [10].
A way to characterize the shape of a polymer is the gyration tensor. It is defined by

$$S_{mn} = \frac{1}{N} \sum_{i=1}^{N} r_m^{(i)} r_n^{(i)} \tag{1}$$

Here $\mathbf{r}^{(i)}$ is the coordinate vector of the ith monomer and the subindex denotes its cartesian components. The eigenvalues $\lambda_1 \leq \lambda_2 \leq \lambda_3$ give the squared lengths of the principal axes of gyration. The ratios of the eigenvalues indicate the deviation from a sphere-like shape of the polymer. The gyration tensor is illustrated in a 2D sketch in Fig. 6A for an elongated as well as a compact polymer, yielding a pronounced difference in the ratio of the tensor's principal axes. While in an averaged sense, polymers display an isotropic behavior, single conformations are markedly aspherical [11,12]. The self-avoiding walk, for example, has averaged eigenvalue ratios of $\langle\lambda_3\rangle : \langle\lambda_2\rangle : \langle\lambda_1\rangle = 14 : 2.98 : 1$.
Typical chromosome conformations are shown in Fig. 6B for different looping probabilities. In Fig. 6C the ratios $\langle\lambda_3\rangle \,/\, \langle\lambda_1\rangle$ and $\langle\lambda_2\rangle \,/\, \langle\lambda_1\rangle$ between the principal axes of the chromatin model are shown. Clearly, in the range of loop numbers where leveling-off occurs, the shape of the polymer is such that it is more elongated in one direction by a factor of $\sqrt{2}$ to $\sqrt{5}$. As for the distance fluctuations, this is in contrast to compact globular polymers that have a spherical shape, but in agreement with experimental [6] and simulational studies [13,14].

References

[1] Cremer T, Cremer C (2001) Chromosome territories, nuclear architecture and gene regulation in mammalian cells. Nat Rev Genet 2: 292–301.

[2] Meaburn KJ, Misteli T (2007) Cell biology: Chromosome territories. Nature 445: 379–381.

[3] Bolzer A, Kreth G, Solovei I, Koehler D, Saracoglu K, et al. (2005) Three-dimensional maps of all chromosomes in human male fibroblast nuclei and prometaphase rosettes. PLoS Biol 5: e157.

[4] Cremer T, Cremer C, Schneider T, Baumann H, Hens L, et al. (1982) Analysis of chromosome positions in the interphase nucleus of chinese hamster cells by laser-UV-microirradiation experiments. Hum Genet 62: 201–209.

[5] Goetze S, Mateos-Langerak J, Gierman HJ, de Leeuw W, Giromus O, et al. (2007) The three-dimensional structure of human interphase chromosomes is related to the transcriptome map. Mol Cell Biol 27: 4475–4487.

[6] Khalil A, Grant J, Caddle L, Atzema E, Mills K, et al. (2007) Chromosome territories have a highly nonspherical morphology and nonrandom positioning. Chromosome Res 15: 899–916.

[7] Chandley AC, Speed RM, Leitch AR (1996) Different distributions of homologous chromosomes in adult human sertoli cells and in lymphocytes signify nuclear differentiation. J Cell Sci 109 (Pt 4): 773–776.

[8] Comings DE (1980) Arrangement of chromatin in the nucleus. Hum Genet 53: 131–143.

[9] Sun HB, Shen J, Yokota H (2000) Size-dependent positioning of human chromosomes in interphase nuclei. Biophys J 79: 184–190.

[10] Eils R, Dietzel S, Bertin E, Schröck E, Speicher MR, et al. (1996) Three-dimensional reconstruction of painted human interphase chromosomes: active and inactive X chromosome territories have similar volumes but differ in shape and surface structure. J Cell Biol 135: 1427–1440.

[11] Šolc K, Stockmayer WH (1971) Shape of a random-flight chain. J Chem Phys 54: 2756-2757.

[12] Bruns W (1992) Angular-correlations within coiled polymer-molecules. Makromolekulare Chemie-Theory And Simulations 1: 287–293.

[13] Rosa A, Everaers R (2008) Structure and dynamics of interphase chromosomes. PLoS Comput Biol 4: e1000153.

[14] Cook PR, Marenduzzo D (2009) Entropic organization of interphase chromosomes. J Cell Biol 186: 825–834.

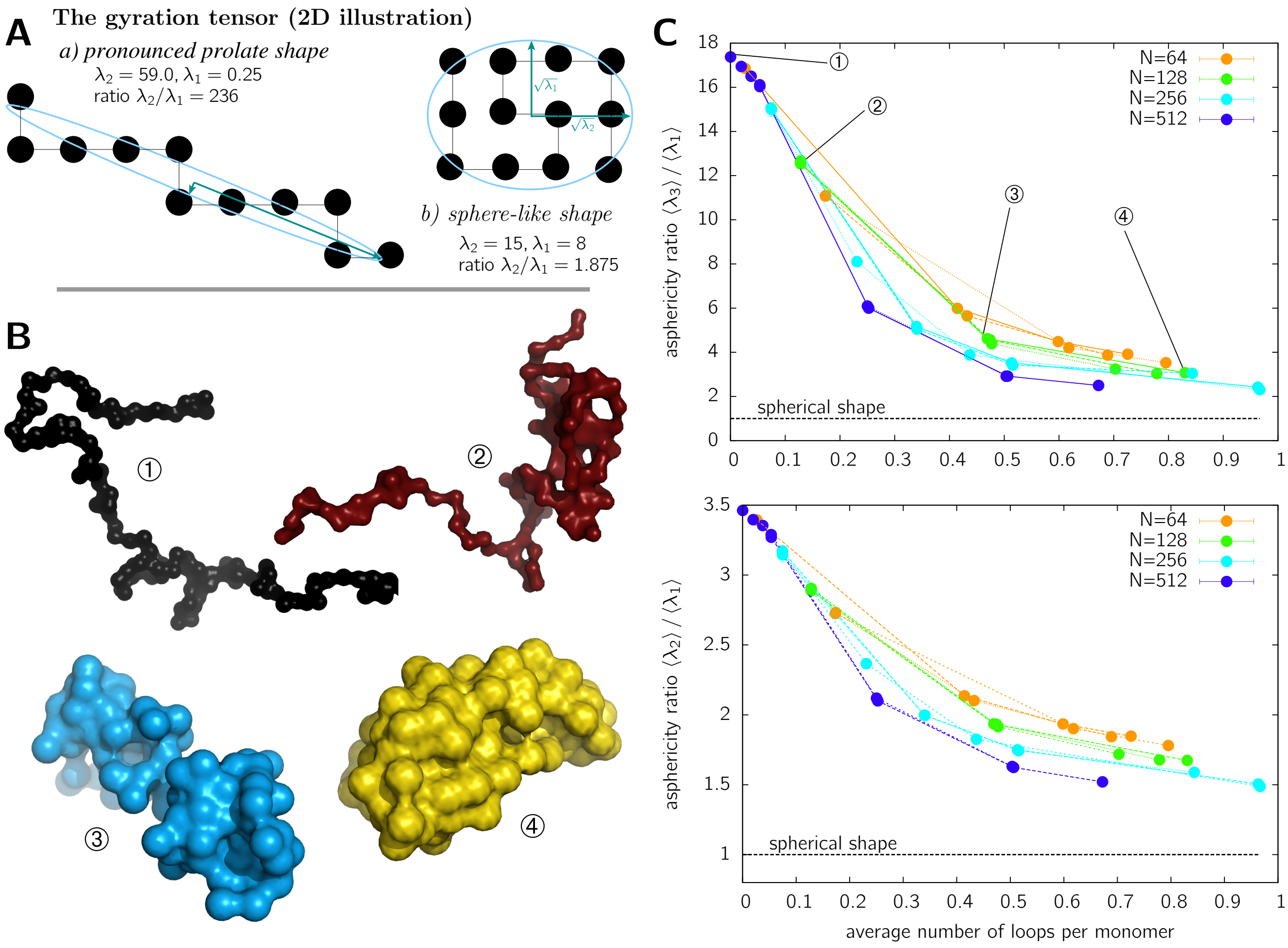

Figure 6: **Elongated shape of the chromatin model polymers. A.** Illustration of the gyration tensor. The gyration ellipsoid is shown for an elongated and a compact polymer conformations in two dimensions. The ratio λ_2/λ_1 is large for the elongated polymer, indicating strong devations from a sphere-like shape. **B.** Example conformations for a chain of length $N = 128$ and loop lifetime τ_1 (see eq. **??**) for different looping probabilities. The shown conformations are one sample of the ensemble of conformations belonging to the data point marked in figure C. **C.** The ratios between the gyration tensor's main axes. The upper graph shows the ratio between the largest and smallest main axis, the lower graph the ratio between the second largest and smallest main axis. The data is shown for chain length up to $N = 512$, different lifetimes of the loops ($\tau = \tau_1$ solid line, $\tau = \tau_2$ dotted line, $\tau = \tau_3$ dashed line) and different looping probabilities p.
